# Surface topography regulates wnt signaling through control of primary cilia structure in mesenchymal stem cells

**DOI:** 10.1038/srep03545

**Published:** 2013-12-18

**Authors:** R. J. McMurray, A. K. T. Wann, C. L. Thompson, J. T. Connelly, M. M. Knight

**Affiliations:** 1 Institute of Bioengineering and School of Engineering and Materials Science, Queen Mary University of London, Bancroft Road, Mile End, London E1 4NS, UK; 2 Institute of Bioengineering and Institute of Cellular and Molecular Sciences, Blizard Institute, Barts and The London School of Medicine and Dentistry Queen Mary University of London, 4 Newark Street London, E1 2AT, UK

## Abstract

The primary cilium regulates cellular signalling including influencing wnt sensitivity by sequestering β-catenin within the ciliary compartment. Topographic regulation of intracellular actin-myosin tension can control stem cell fate of which wnt is an important mediator. We hypothesized that topography influences mesenchymal stem cell (MSC) wnt signaling through the regulation of primary cilia structure and function. MSCs cultured on grooves expressed elongated primary cilia, through reduced actin organization. siRNA inhibition of anterograde intraflagellar transport (IFT88) reduced cilia length and increased active nuclear β-catenin. Conversely, increased primary cilia assembly in MSCs cultured on the grooves was associated with decreased levels of nuclear active β-catenin, axin-2 induction and proliferation, in response to wnt3a. This negative regulation, on grooved topography, was reversed by siRNA to IFT88. This indicates that subtle regulation of IFT and associated cilia structure, tunes the wnt response controlling stem cell differentiation.

Primary cilia are expressed by most mammalian cell types and exist as a singular cytosolic compartment, often projected into the extracellular environment. They consist of a tubulin scaffold wrapped in a specialized section of the plasma membrane. Increasingly, the cilium has been implicated in various aspects of cell biology including regulation of the cell cycle, migration and the cellular response to external stimuli such as hedgehog, wnt, growth factors, inflammatory cytokines and mechanics^1^,^2^,^3^,^4^,^5^,^6^,^7^,^8^,^9^,^10^,^11^,^12^,^13^. By these means the cilium exerts influence in tissue homeostasis and pathology but is also involved in tissue development and stem cell differentiation. Ciliogenesis occurs upon growth arrest and entry of the cell into G0. Extension takes place from the basal body, a structure modified from one of the centrioles, hence ciliogenesis and cell division are mutually exclusive and inextricably related. Intraflagellar transport (IFT) is required for ciliogenesis, conducting traffic into and out of the cilium and ultimately supporting cilia function^14^,^15^. The increasing number of identified ciliopathies that stem from changes in cilia length as a result of mutations in IFT, suggest that IFT plays a key role in the relationship between cilia structure and its function as a signaling hub^16^,^17^,^18^,^19^,^20^,^21^.

The primary cilium is well established as the location for the transduction of ligand-induced hedgehog pathway activation^22^ but the canonical and non-canonical wnt signaling pathways have also been proposed to be regulated by the cilium. In particular, ciliogenesis itself is thought to induce a switch from canonical signaling towards non-canonical wnt signaling. Lancaster *et al.*, recently identified that the primary cilium accomplishes negative regulation of canonical wnt signalling by sequestering β-catenin away from the nucleus by means of the protein Jouberin, also the mediator of β-catenin nuclear entry. In doing so, the cilia compartment brings about a reduction in the cells responsiveness to wnt3a^6^. Additionally other cilia-localised proteins such as glycogen synthase kinase (GSK) and inversin have been shown to negatively regulate β-catenin signaling, with inversin acting as a switch inhibiting canonical wnt signaling whilst activating non-canonical wnt signaling^23^,^24^. As canonical Wnt signaling is classically considered to promote proliferation whilst non-canonical wnt signaling is thought to promote differentiation, this may represent a mechanism through which the primary cilium can regulate stem cell differentiation. Indeed, the primary cilium has been shown to function in various types of stem cell differentiation, including cardiomyocyte, neural and mesenchymal, with several studies identifying cilia presence and length as crucial factors for differentiation^1^,^3^,^25^,^26^,^27^,^28^. Recently, Hoey *et al.*, demonstrated a role for the primary cilium in mediating a mechanotransduction response to oscillatory fluid flow resulting in an increase in osteogenic differentiation of MSCs^29^. *In vitro* increases in cilia length have been attributed to various factors including a reduction in intracellular calcium, alterations in cyclic AMP and the signaling pathways activated on reception of growth factors and inflammatory cytokines^7^,^8^,^17^,^30^. However, the actin cytoskeleton^18^,^31^ and actin modulated intracellular tension has recently been identified as another modulator of cilia length, with decreased actin-myosin contractility found to increase cilia length and prevalence^32^.

Surface topography-mediated changes in intracellular tension has been widely documented to regulate both the differentiation and self-renewal of MSCs^33^,^34^,^35^. Indeed, the reciprocal relationship between cell shape and intracellular tension is known to affect various cellular processes involved in differentiation including proliferation cell signaling. Micro- and nanotopography produced using techniques more commonly employed in the electronics industry have been used to study the effect of cell adhesion on intracellular tension and stem cell differentiation^36^,^37^,^38^,^39^,^40^. Other techniques such as micro-contact printing produce surfaces that also regulate intracellular tension through changes in whole cell shape and adhesion^41^,^42^,^43^,^44^.

Previous studies by Kilian *et al.*, observed that MSCs under increased cellular contractility and intracellular tension are more susceptible to soluble factors. We propose that in the case of wnt signaling this is due to an alteration in cilia prevalence and length, both functions of cilia trafficking, and which is also regulated by actin organization and contractility. Therefore this study tests the hypothesis that grooved topographic surfaces induce a cell shape mediated decrease in intracellular actin-myosin tension, which leads to an increase in primary cilia length and prevalence and an associated alteration in wnt signaling.

We show that primary cilia respond to reduced actin cytoskeletal organization induced by surface topography with subtle, but statistically significant, elongation. Using a ROCK inhibitor to prevent actin stress fiber formation on the flat surface, primary cilia length was found to increase to lengths comparable to those measured on untreated grooved surfaces. Reductions in cilia length with siRNA to IFT88 increased nuclear β-catenin localization, a marker for canonical wnt signaling response. Similarly nuclear β-catenin localization was lower in MSCs on flat surfaces with shorter cilia compared with cells on grooved topography. The response to grooves was prevented when cilia structure was disrupted using siRNA-IFT88. In addition grooved topography abolished downstream axin-2 induction and proliferation in response to wnt3A. This study not only adds to the growing body of evidence for the importance of the cilium in stem cell biology, but highlights how subtle alterations in ciliogenesis, in this case induced by topography regulate wnt signalling, a role particularly crucial within the stem cell niche.

## Results

### Substrate topography directs primary cilia orientation

**Figure 1:**
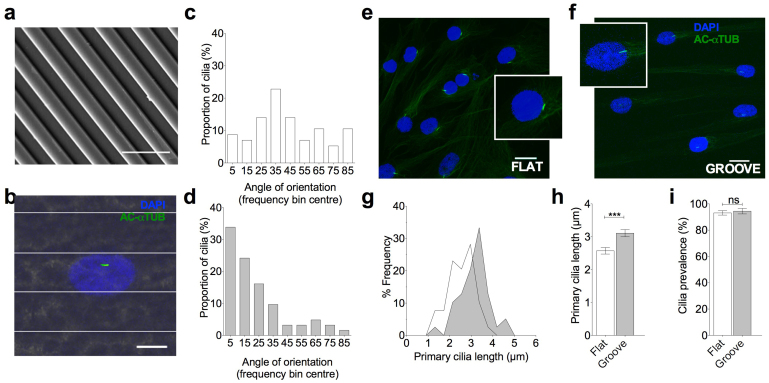
Stem cell primary cilia orientate parallel to substrate grooves and elongate compared with flat culture. (a) SEM image of grooved substrate. Scale bar represents 10 μm. (b) Representative immunofluorescent confocal maximum projection image of MSCs cultured on grooved substrate. The primary cilia are labeled with anti-acetylated alpha tubulin (green) and nuclei counterstained with DAPI (blue). Scale bar represents 15 μm. Fluorescent image overlaid on reflectance image to enable marking of groove ridges. (c/d) Frequency distributions showing the angle of orientation of the primary cilia on flat and grooved substrates respectively. (e/f) Confocal 3D maximum projection images showing primary cilia in cells grown on flat and grooved surfaces respectively. Inserts showing primary cilia at 4 × higher magnification. Cells were labeled with anti-acetylated alpha tubulin (green) and nuclei counterstained with DAPI (blue). Scale bar represents 10 μm. (g) Frequency histogram for primary cilia length on flat and grooved surfaces (dark grey is the grooved surface).(h) Effect of topography on primary cilia length. Values represent mean with error bars indicating SEM. (i) Effect of topography on primary cilia prevalence with error bars indicating S.E.M.

To investigate the effect of microtopography on the structure of the primary cilium, MSCs were cultured on microgrooves (Figure 1a) and a flat control surface for a period of 24 hrs in the presence of serum. This was followed by 24 hrs serum starvation to induce cell cycle arrest and to allow stable ciliogenesis. Primary cilia were identified using immunocytochemistry, labeling acetylated alpha-tubulin (Figure 1b). MSCs cultured on the microgrooves were found to adopt an elongated morphology, orientated in the direction of the grooves but not contained by the grooves (Figure 1b). MSCs cultured on flat surfaces expressed randomly orientated primary cilia and thus produced the predictable even distribution for angle of orientation (Figure 1c). By contrast the primary cilia of MSC cultured on grooves were found to preferentially align parallel to the grooves with a median angle of orientation of 17 degrees compared with 39 degrees on the flat, the difference being statistically significant (p < 0.0001, n = 62 and 57 cilia respectively) (Figure 1d).

### Topography increases primary cilia length

MSCs cultured on the grooved surfaces and subjected to 24 hrs serum starvation, were found to have increased primary cilia length when compared with those on flat surfaces, the difference being statistically significant (p < 0.0001, n = 109 and 102 cilia respectively from 3 experiments). The majority of MSCs on the grooves had primary cilia with lengths greater than 3 μm, with an overall increase in average length of 26% with respect to flat (Figure 1e–h). The angle of cilia relative to the horizontal substrate was also measured and used to estimate the true length of each cilium in 3D (see supplementary figures S1 and S2). This analysis confirmed that the increase in projected length is not simply due to flattening of cilia onto the substrate. Cells were quiescent, as indicated by rarity of ki67 positive staining to identify cell cycle status in these serum starved preparations. Predictably therefore, cilia prevalence was very high. MSCs displayed over 95% ciliation on both the grooved and flat surfaces, with no statistically significant difference observed (Figure 1i). The changes in cilia length associated with the topography were therefore not due to changes in cell cycle and resultant differential ciliogenesis. These data demonstrate that culturing MSCs on grooved substrates produces a significant increase in the length of primary cilia without modulation of cell cycle or cilia prevalence.

### Grooved topography regulates primary cilia length via ROCK-dependent changes in actin

**Figure 2:**
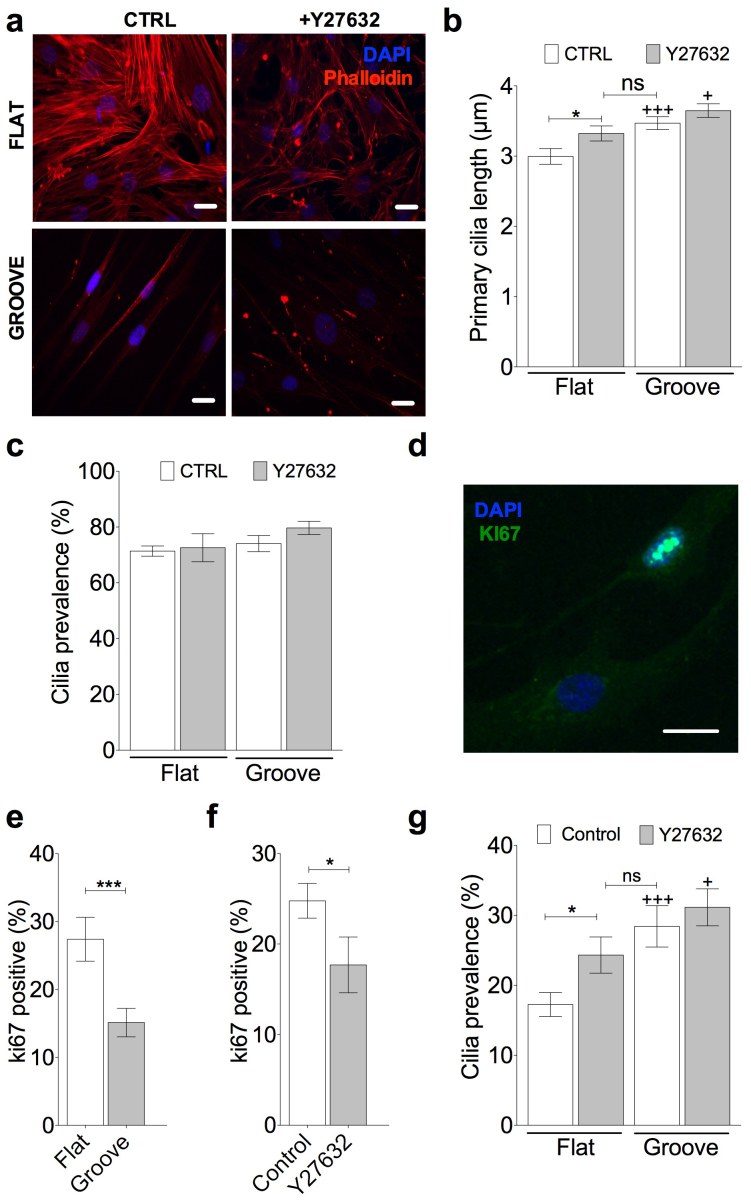
Rho/Rock dependent actin modulation governs topography-induced changes in primary cilia structure. (a) Alexa Phalloidin staining for f-actin (red) and nuclei stained with Dapi (blue) for MSCs cultured on flat and grooved surfaces with and without the Rho/ROCK inhibitor Y27632. Scale bar represents 10 μm. (b) Primary cilia length data for flat and grooved surfaces with and without Y27632 treatment. (+) represent statistical differences with respective flat group. (c) Primary cilia prevalence data for flat and grooved surfaces with and without Y27632 treatment. (d) representative ki67 staining showing one positive and one negative cell. Scale bar represents 10 μm. Prevalence of Ki67 positive staining and the influence of (e) surface topographies and (f) Rho/Rock inhibition by Y27632. (g) Primary cilia prevalence, with and without Rho/Rock inhibition with Y27632, on both topographies. Statistically differences denoted by + symbols are relative to respective flat topography group.

We next examined the actin cytoskeleton in MSCs cultured on grooved and flat topographies as studies have identified actin organisation as a regulator of primary cilia length. Micro-contact printed surfaces have been shown to increase cytoskeletal tension and inhibit primary cilia extension whilst surfaces that lead to a reduced cytoskeletal tension result in longer cilia^32^. We used alexa 555-conjugated phalloidin to stain for F-actin and a Rho kinase inhibitor Y27632 (10 μM) to inhibit the formation of actin stress fibers in MSCs cultured on flat and grooved surfaces. In the absence of Y27632, MSCs cultured on the flat surfaces formed well-defined actin stress fibers. However these features were much less distinct for cells cultured on grooved surfaces (Figure 2a). Treatment with Y27632 was observed to disrupt the formation of actin stress fibers on both the grooved and flat surfaces. In agreement with a role for actin cytoskeleton, a significant increase in average primary cilia length was found to occur on the flat surface treated with Y27632 (p = 0.012, n = 92 and 85 cilia respectively from 3 experiments). Indeed this increase in length was such that cilia length was not-significantly different compared with untreated MSCs cultured on the grooves (Figure 2b). Y27632 treatment did not abolish the statistically significant changes in cilia length between flat and grooved topography (p < 0.0001). Treatment with Y27632 was not found to significantly alter cilia prevalence across the surfaces (Figure 2c). Thus topography-induced changes in cilia length require actin remodeling.

### Topography and actin cytoskeleton regulate cell cycle re-entry and primary cilia prevalence in the presence of serum

Having shown that topography modulates primary cilia length, presumably through the control of IFT and associated cilia trafficking, we wished next to examine whether this influences MSC responses to potential differentiating stimuli. For *in vitro* differentiation protocols, MSC's are normally grown in the presence of serum, a prominent regulator of cell cycle re-entry. Therefore it is important to examine the role of the cilium under conditions without serum starvation. In the presence of serum, cilia length was not assessed due to the more dynamic nature of cilia assembly and disassembly in these cultures. Furthermore, cilia lengths were generally found to be < 0.5 μm making accurate measurement difficult. However, the presence of the primary cilium (prevalence) was assessed as both length and prevalence are ultimately governed by ciliary trafficking.

Under serum conditions, many MSCs cultured on both flat and grooved surfaces exhibited ki67 positive cells as exemplified in Figure 2d, indicating the induction of cell cycle re-entry. Furthermore, cultures on grooved substrates were found to have approximately half the percentage of ki67 positive cells as that found on flat surfaces (Figure 2e), the difference being statistically significant (p = 0.001, n = 26 and 29 fields respectively, a total of at least 250 cells for each condition taken from 3 experiments). This indicates that the grooves reduce cell proliferation with respect to flat controls. Similarly a reduction in actin organization by Y27632 also negatively affects cell cycle re-entry compared with untreated controls (p = 0.03, n = 21 fields for each, a total of at least 200 cells for each condition taken from 2 experiments) (Figure 2f). Change in topography is associated with a statistically significant difference in primary cilia prevalence in the presence of serum (Figure 2g) with mean values of 30% on grooved and 18% on flat substrates (p = 0.0008, n = 25 and 31 fields each, a total of at least 200 cells for each condition taken from 2 experiments). It appears that, as with serum starved cultures, this is also regulated by actin cytoskeleton remodeling. Consequently Y27632 increases cilia prevalence on flat surfaces, a statistically significant effect (p = 0.039, n = 84 and 91 cells respectively from at least 3 experiments) such that the value is not significantly different from that found for untreated cells on grooved surfaces (Figure 2g). Inhibition of actin remodeling does not completely mask the effect of grooved topography on cilia prevalence. A statistically significant difference still exists between flat and grooved surfaces treated with Y27632 (p = 0.04, n = 91 and 87 cells respectively from at least 3 experiments). These data therefore demonstrate that topography modulates primary cilia structure in both cycling and non-cycling conditions through modulation of actin organization and cell tension. However, in serum cultures the cell cycle is the dominant factor regulating ciliogenesis.

### Disruption of the cilium using IFT88 siRNA enhances MSC response to wnt3a

**Figure 3:**
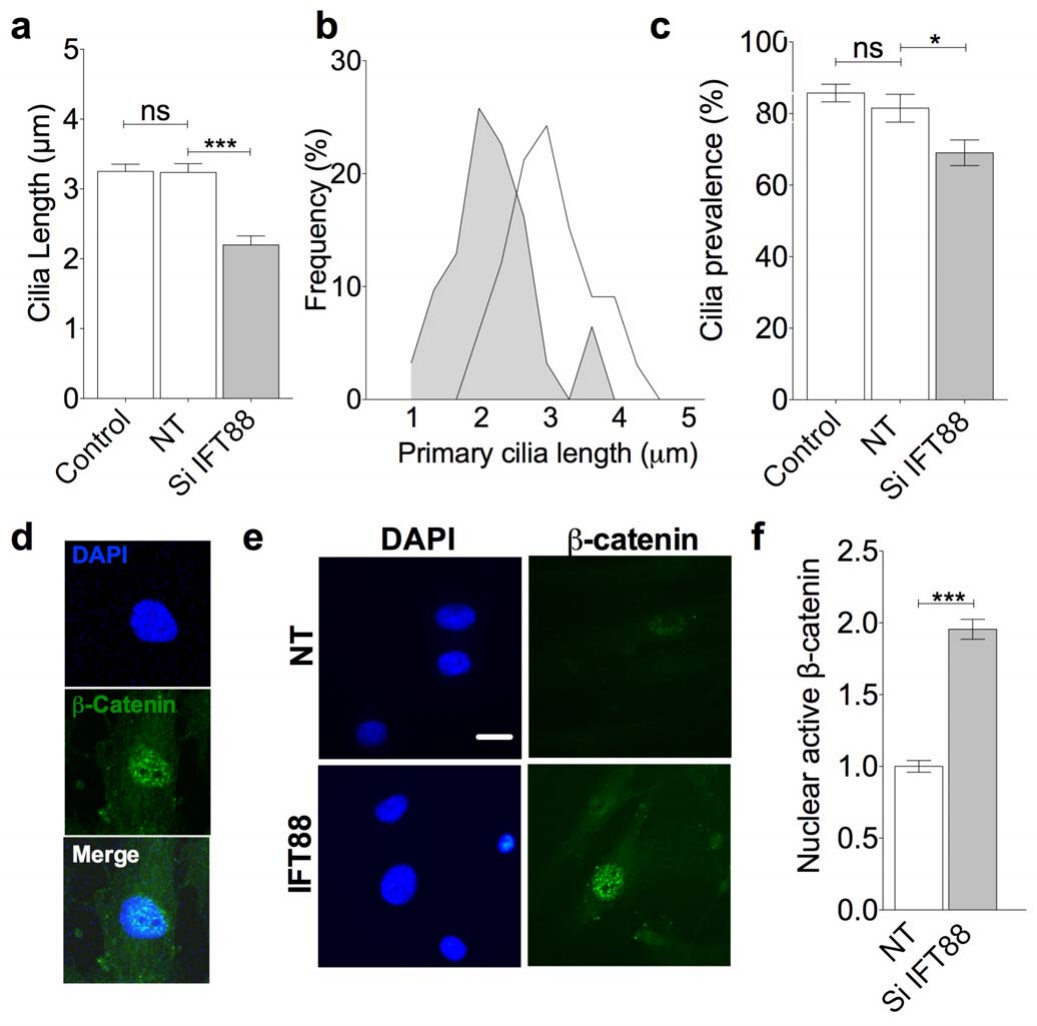
Negative modulation of cilia assembly and trafficking by IFT88 siRNA increases canonical wnt signaling in response to wnt3a. (a) Primary cilia length assessed in control preparations and those transfected with a non-targetting (NT) siRNA pool and a pool directed to IFT88. (b) Frequency histogram for siRNA treatment effect on primary cilia length (dark grey is siRNA to IFT88, clear is non-targetting. (c) Primary cilia prevalence with IFT88 siRNA transfection. (d) Representative images showing nuclear active β-catenin with wnt3a treatment. Nuclear masks were drawn around DAPI signal. Scale bar represents 10 μm. (e) Representative images showing nuclear active β-catenin in non-targeted and siRNA IFT88 cells. Scale bar represents 10 μm. (f) Quantitative analysis of relative nuclear active β-catenin expression with wnt3a treatment in non-targeted and IFT88 siRNA targeted cells on flat surface.

To investigate the influence of primary cilia structure in regulating wnt signaling we assessed wnt signaling following disruption of intraflagellar transport mediated ciliary assembly by means of IFT88 (anterograde IFT particle) directed siRNA transfection. Previously, mutations of retrograde intraflagellar associated motors, such as retrograde dynchc2, showed ability to modulate wnt signaling including at the level of β-catenin translocation to the nucleus^6^. This is proposed to be because the cilium sequesters β-catenin using IFT, at the expense of nuclear entry. First an appropriate dose of siRNA was established such that transfection resulted in a partial, negative regulation of primary cilia assembly characterized by a reduction in cilia length. Quantification of the magnitude of effect these transfections had on IFT88 gene expression was assessed by qPCR and shown in supplementary figure S3. Transfections statistically significantly reduced IFT88 message on flat and grooved topographies, 50% and 60% respectively. This is a partial knock-down of IFT88 at lower doses than required for complete disruption of ciliogenesis. Non-targeted siRNA had no statistically significant effect on primary cilia length or prevalence with respect to non-transfected cells. By contrast, IFT88 directed siRNA produced a statistically significant difference in cilia length with a reduction in average length of 35% compared with non-targeting siRNA transfected cells (p = < 0.0001, n = 72 and 83 cilia respectively from 3 experiments, Figure 3a). Length changes were seen through the breadth of the population as indicated by a shift towards shorter cilia depicted in the histogram for cilia length in Figure 3b. This was also associated with a 20% reduction in primary cilia prevalence from a mean of 85.8% with non-targeted siRNA to 69.0% in IFT88 siRNA targeted cells (p < 0.025, n = > 200 cells for each condition from 2 experiments, Figure 3c). No obvious changes to actin organization were observed with siRNA treatment. Canonical wnt signaling in response to addition of wnt3a was assessed by quantification of active β-catenin located in the nucleus on a cell by cell basis (Figure 3d). IFT88 siRNA transfection, using cells on flat topography, resulted in a near doubling of active β-catenin levels in the nucleus. This increase was statistically significant (p < 0.0001, n = 48 and 54 cells respectively from 3 experiments (Figure 3e and f)). This indicates that reductions in primary cilia length and cilia prevalence up-regulate canonical wnt signaling.

### Cellular response to wnt3a is down-regulated in MSCs cultured on grooves

**Figure 4:**
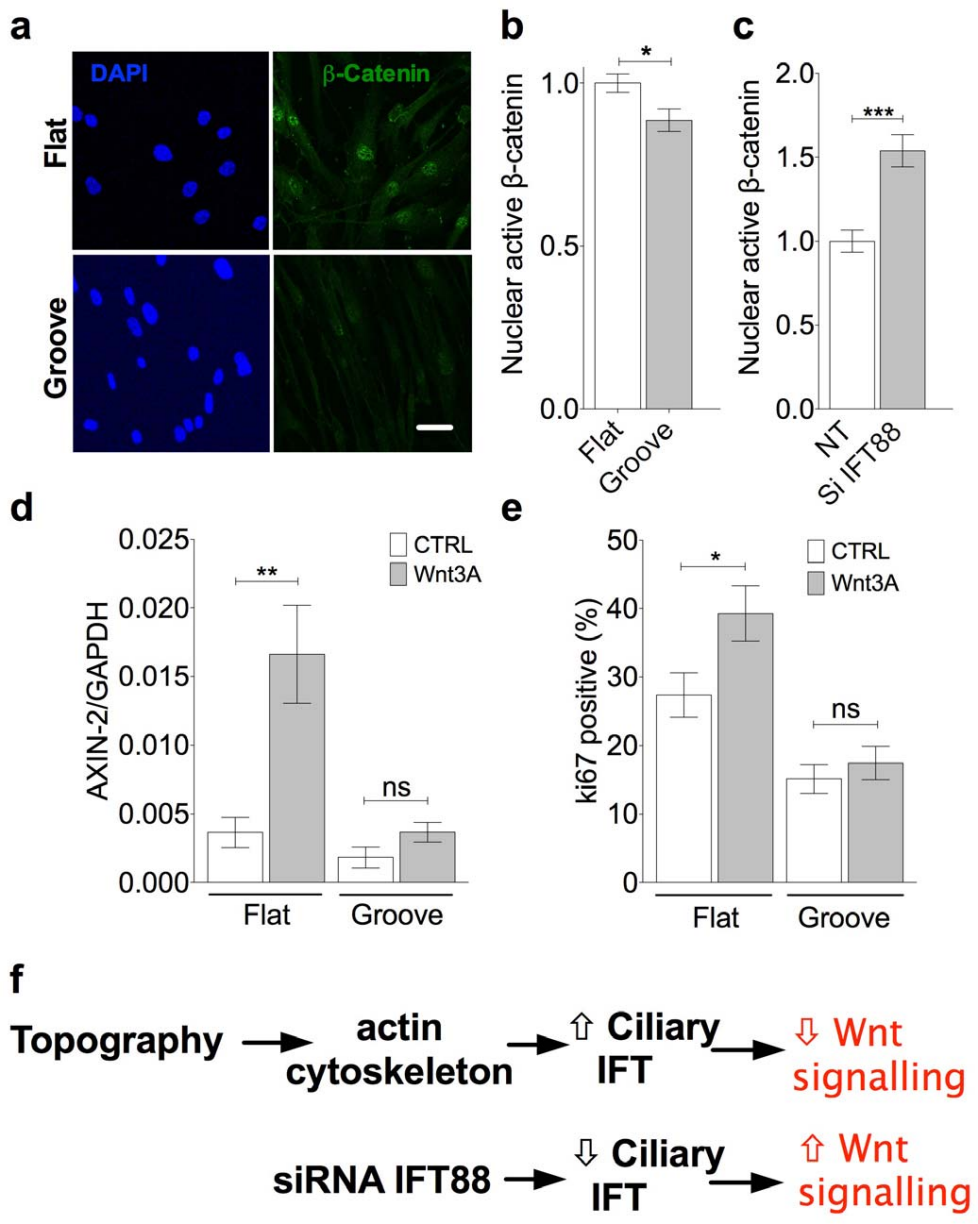
Topography influences canonical wnt signaling response to wnt3a. (a) Representative fluorescence images showing nuclear active β-catenin localization with wnt3a treatment on flat and grooved surfaces. Scale bar represents 10 μm. (b/c) Quantitative analysis of relative active nuclear β-catenin with wnt3a treatment. (b) The effect of surface topography with values normalized to mean value for cells on flat surfaces. (c) The effect of IFT88 siRNA for cells on grooved surfaces with values normalized to non-targeting control. (d) Quantitative analysis of relative axin-2 expression normalized to GAPDH with wnt3a treatment. (e) Ki67 positive staining to assess the proliferative response to wnt3A, on both topographies. (f) Schematic of proposed summary of findings.

We finally sought to establish the effect of the grooved topography on canonical wnt signaling and whether this required the increase in primary cilia assembly shown to occur through reduced cytoskeletal tension. This was initially indicated by the intensity of active β-catenin localization in the nucleus but also supplemented by assessment of axin-2 gene expression, another canonical wnt marker. The topographical microenvironment affected the response to wnt3a such that active β-catenin levels in the nucleus were statistically significantly lower on grooves with a 12% reduction in mean staining intensity compared with that on flat surfaces (p = 0.0125, n = 45 and 39 cells respectively from 2 experiments, Figure 4a and b). siRNA disruption of IFT88 produced a similar whole population shift towards shorter cilia lengths, as seen for flat surfaces (siRNA efficacy shown in supplementary figure S3). siRNA disruption of IFT88, in cells cultured on grooves, increased active β-catenin levels in the nucleus thus reversing the negative effects of topography (p < 0.0001, n = 54 and 51 cells respectively from 2 experiments, Figure 4c). Down-regulation of wnt signaling by grooved topography relative to flat is also observed when downstream gene expression marker axin-2 was assessed in response to wnt3A. The statistically significant increase in axin-2 expression with wnt3a treatment on flat surfaces is abolished on grooved topography (p = 0.0087, paired t-test, n = 6, Figure 4d). Similarly wnt3a-induced cell cycle re-entry, as assessed by ki67 staining, is also abolished by culture on grooves (4e). Importantly, and in concord with these cell cycle findings, wnt3a treatment has no effect on cilia prevalence on grooved topography. These results are consistent with the proposal that grooved topography reduces cytoskeletal tension and cell cycling. This in turn promotes ciliogenesis or elongation by intraflagellar transport and promotes the negative regulation of wnt signalling by the cilium (Figure 4f). This can be reversed by negative regulation of anterograde IFT with siRNA to IFT88.

Thus the data supports the hypothesis that subtle changes in cilia structure, as modulated here by topography, regulate wnt responsiveness. Thus our data indicates that stem cell canonical wnt signaling in response to wnt3a is reduced on grooved surfaces due to increased primary cilia assembly and trafficking.

## Discussion

In recent years mesenchymal stem cells have been shown to be highly responsive to their physical environment, with physical, topographic cues having been shown to support and induce *in vitro* stem cell self-renewal and differentiation respectively. The accessibility and multipotent potential of MSCs makes them an ideal target for use in regenerative therapies. However, for MSCs to play a key role in regenerative applications an understanding of the pathways via which these cues affect stem cell differentiation is critical. This information will also aid the understanding of fundamental stem cell differentiation *in vivo*.

The culture of MSCs on a microgrooved surface topography was found to promote alignment of the cells parallel to the grooves. Many studies have shown similar contact guidance of various cell types in response to topographic cues. However, this is the first study to show alignment of the primary cilium in response to topography which may influence planar cell polarity and directional migration. The topography used here does elicit changes to gross cell shape but the cells are not confined to grooves and often track to adjacent grooves. Despite this a high degree of cilia orientation to grooves is observed. Primary cilia orientation has been reported in response to chemical cues, driving cell polarity and migration^2^. The orientation of primary cilia also occurs in tendon where the cilia align parallel to the predominant collagen orientation^45^. In both cases cilia orientation may be associated with actin organization and integration with the cilium basal body although the precise mechanisms are unclear.

Cilia length has previously been shown to undergo regulation by multiple factors including various signaling cascades, inflammatory signals, the cytoskeleton^18^,^31^ and more recently cell shape. Pitaval *et al.* used microcontact printing to regulate cell shape, and identified that when cells were allowed to form strong intracellular actin networks, cilia length and prevalence were reduced^32^. In contrast, cilia were significantly longer in cells that were unable to form well defined actin networks. Here, we have shown that topography-induced changes in cell shape lead to a significant increase primary cilia length. Previous studies have linked cytoskeletal actin tension to regulating the differentiation and proliferation of stem cells^34^. Using a pharmacological inhibitor of ROCK, Y27632, we show that reducing actin stress fiber formation directly results in cilia elongation without altering cilia prevalence. These results are therefore in agreement with those reported by Pitaval *et al.* confirming the role of actin tension in regulating cilia structure. We also go on to show that in serum conditions, where the cell cycle is a factor, actin remodeling continues to exert an influence on cilia prevalence as a result of cell cycle re-entry.

To demonstrate the role of the primary cilium, and specifically cilia assembly, in regulating canonical wnt signaling in MSCs, an siRNA for IFT88 was used to negatively regulate cilia assembly, a sub-optimal effect designed to partially reduce IFT88 activity. Ciliary trafficking has been previously shown to be at the heart of the partial modulation of wnt signalling, itself an important regulator of stem cell differentiation^46^. IFT drives the ciliary sequestration of β-catenin at the expense of nuclear entry by means of ciliary localised Jouberin^6^. In contrast to previous work investigating the role of the cilium in signaling, length changes with topography were subtle (25% increase). We therefore used an appropriate IFT88 siRNA treatment such that the magnitude of effect was of a similar order (35% reduction). In response to treatment with wnt3a ligand, MSCs treated with an IFT88 targeted siRNA pool were found to have markedly increased active nuclear β-catenin compared to a non-targeting control.

We show that changes in primary cilia assembly, mediated by topography and cytoskeletal tension, result in changes in the influence the cilium has over canonical wnt signaling at the level of β-catenin accumulation in the nucleus but also more dramatically at the level of downstream axin-2 gene expression. MSCs cultured on the grooved surface were found to be less responsive to treatment with wnt3a such that nuclear active β-catenin expression was diminished, the induction of axin-2 gene expression abolished and proliferation reduced. This down-regulation on grooved topography was reversed with siRNA targeted to IFT88 consistent with the proposals of Lancaster *et al*. that primary cilia trafficking sequesters β-catenin.

These results indicate that both positive and negative regulation of cilia assembly can finely tune MSC responsiveness to wnt stimulation in opposing directions. Furthermore, given the role of the primary cilium in other signaling pathways, in particular the hedgehog and PDGF signaling, it is likely that topography-induced changes in cilia structure may regulate these pathways which also have an involvement in stem cell differentiation. In conclusion, this study demonstrates for the first time, that topographical cues regulate wnt responsiveness through alterations in primary cilia structure as a result of differential cytoskeletal tension. Alterations in cilia assembly lead to changes in how cells respond to wnt3a stimulation, switching cells from the canonical to the non-canonical pathway. The results highlight the possibility of manipulating wnt signaling and associated stem cell differentiation through control of primary cilia assembly and trafficking. This may be achieved using biomaterials incorporating topographical cues or direct pharmaceutical intervention. This approach has important potential applications in tissue engineering and regenerative medicine and may also be applicable to other cell types and diseases involving wnt signaling.

## Methods

### Surface preparation

Quartz slides with 12.5 μm wide, 0.54 μm deep grooves were produced using photolithography as described in^44^. Polycaprolactone (PCL) (Sigma, UK) beads were melted at 70°C and micro-grooves manually embossed into the polymer using the micro-grooved quartz slide and a flat quartz slide for controls. Topographically patterned PCL was examined by SEM (Figure 1a) which confirmed that that the dimensions accurately matched the quartz slide.

### Cell culture

Surfaces were first sterilised under UV light for 20 min, then coated in fibronectin (10 μg.mL, Millipore, UK) to increase cell adhesion followed by two sequential washes in PBS. Human bone marrow-derived mesenchymal stem cells (MSCs) (Lonza, UK) were seeded onto the grooved and planar surfaces at 1.25 × 10^4^ cells/cm^2^. Cells were cultured for 24 hours in basal media (α-MEM (PAA, UK) supplemented with 10% FBS and Penicillin-Streptomycin followed by 24 hours serum starvation to induce ciliogenesis. For activation of canonical wnt signaling, wnt3a (150 ng.mL, Peprotech, UK) was added to serum-free media for 3 hours following serum starvation. A ROCK inhibitor (Y27632, 10 μM, Sigma, UK) was added to the serum-free media 24 hours after cell seeding to biochemically inhibit actin stress fibre formation.

### Immunocytochemistry

Cells were fixed in 4% paraformaldehyde for 10 min at 37°C and permeabilized in 0.5% triton X in phosphate buffered saline (PBS, Sigma, UK) for 5 min at room temperature. Non-specific binding sites were blocked by incubating cells for 30 min in 5% goat serum in PBS at room temperature. The samples were then washed in 0.1% bovine serum albumin in PBS before incubation with the appropriate primary antibody. Samples were washed a further three times in 0.1% PBS/BSA and incubated for 2 hr at room temperature in 0.1% PBS/BSA with the secondary antibody, either anti-mouse 488/555 or anti-rabbit 488/555 (1:500, Sigma, UK). The following primary antibodies were used: mouse anti-acetylated α-tubulin (1:2000, Sigma, UK), mouse anti-active β-catenin (1:500, Millipore, UK), mouse anti-Ki67 (1:500, Sigma, UK) and rabbit anti-pericentrin (1:500, Sigma, UK). In addition F-actin was labeled with alexa 555-conjugated phalloidin (1:50, Molecular Probes, Invitrogen, UK) in 0.1% PBS/BSA overnight at 4°C. All samples were finally washed three times in 1% PBS/BSA and mounted onto glass slides using Prolong Gold mountant containing DAPI.

### Confocal microscopy and measurement of cilia length and orientation

Samples were imaged using a confocal microscope (Leica SP2) with a x63/1.05 NA objective and a pixel size of 0.1 × 0.1 μm. Confocal z-series were made throughout the entire cell monolayer with a z step size of 0.5 μm. Primary cilia lengths and x/y orientation were measured from reconstructed maximum projection images using Image J software as in previous studies^8^. Studies were conducted to determine the angle of cilia orientation relative to the horizontal substrate or z orientation and thus the true cilia length. Data showed that despite a small reduction in angle on grooves true length increases are observed (supplementary figures S1 and S2). The ‘z' Orientation was quantified using the equation outlined in supplementary material, as the smallest angle relative to the axis to the grooves, which were visualized using confocal reflectance microscopy. For MSCs cultured on flat surfaces, primary cilia orientation was measured relative to the horizontal y axis of the image.

### Measuring nuclear β-catenin intensity

Samples were imaged using a fluorescent microscope (Leica) and nuclear β-catenin intensity quantified using Image J software. DAPI staining was used to identify nuclei and to create a mask that was overlaid onto the β-catenin image enabling the mean β-catenin intensity to be calculated for each nucleus. For each group, at least 60 nuclei were assessed from multiple coverslip preparations.

### IFT88 siRNA

MSC's were seeded onto surfaces at 1.25 × 10^4^ cells/cm^2^ in antibiotic-free medium. 24 hrs post seeding cells were transfected as per manufacturers protocol with 25 μM human IFT88 siRNA (ON-TARGET plus smart pool human IFT88, L-012281-01-0005, Dharmacon, USA), a non-targeting siRNA (ON-TARGET plus non-targeting pool, D-001810-10, Dharmacon, USA) or with water as a control using Dharmafect 1 transfection reagent (4 μL/mL) (Dharmacon, USA). Transfection media was replaced after 24 hours and serum free media added for induction of ciliogenesis. After 24 hours serum starvation, 150 ng/mL wnt3a was added for a further 3 hours.

### qPCR

Total RNA was extracted and isolated following the standard protocol associated with Qiagen RNeasy mini kit, quantity and integrity were checked by Nanodrop ND-1000 spectrophotometer (LabTech, East Sussex, UK) and gel electrophoresis analysis. Reverse transcription reactions (including genomic DNA clean up) were performed using Quantitect RT kit (Qiagen). For real time PCR, cDNA was diluted in nuclease free water and triplicate 10 μL reactions were performed using KAPA SYBR® FAST Universal 2X qPCR Master Mix (KAPA Biosystems) containing SYBER-green dye, 0.2 μM ROX reference dye. Optimised primer pairs were as follows axin-2, FOR _GCCAAGTGTCTCTACCTCAT, REV _ACAGGTGATCGTCCAGTATC; GAPDH, FOR _GACAAAATGGTGAAGGTCGG, REV_TCCACGACATACTCAGCACC. IFT88, FOR _ GCAATCCTACGAAACAGTGCC, REV _ CACTGACCACCTGCATTAGC.An annealing temperature of 60°C was used for PCR reactions and fluorescence data was collected using the MX3000P QPCR instrument (Stratagene). Data was analyzed using the relative standard curve method, target genes were normalised to GAPDH expression.

### Statistics and figures

Unless otherwise stated in the text, parametric, non-parametric and contingency statistical tests were used were appropriate and outlined here. For primary cilia length and orientation comparisons non-parametric Mann-Whitney U tests were used as many data sets failed normality testing (Kolmogorov-Smirnov normality test). Primary cilia length data is depicted for the purposes of figures as mean and S.E.M. Prevalence is identically presented but statistical significance was assessed by means of fisher's exact tests using frequency of incidence data for each field. ki67 data is presented as means and S.E.M and also used fisher's exact testing from positive or negative staining in immunofluorescence preparations. Nuclear β-catenin expression is shown relative to the mean of the respective control with figures showing mean and S.E.M values and significant differences assessed using two-tailed unpaired student's t-tests. For gene expression studies two-tailed paired student's t-tests with Bonferoni adjustments to avoid type 1 errors. In all cases statistically significant differences are indicated at p < 0.05 (*), p < 0.01 (**) and p < 0.001 (***). Where important, non-significant differences are labeled ‘ns' on figures. All data analysis and presentation was done using GraphPad prism 5. See text for n numbers.

## Author Contributions

R.J.M., A.K.T.W., J.T.C. and M.M.K. conceived and designed experiments. R.J.M., A.K.T.W. and C.L.T. performed experiments and analysed data. R.J.M., A.K.T.W., C.L.T., J.T.C. and M.M.K. prepared the manuscript. All authors reviewed the manuscript.

## Supplementary Material

Supplementary Information Supplementary material
