# Supplementary material for: Surface topography regulates wnt signaling through control of primary cilia structure in mesenchymal stem cells
Source: Sci Rep. 2013 Dec 18;3:3545. doi: 10.1038/srep03545 (PMC3866595; doi:10.1038/srep03545)
Supplement: Supplementary Information — Supplementary material [file srep03545-s1.pdf]

Surface topography regulates wnt signaling through control of primary cilia structure in mesenchymal stem cells

McMurray R.J.[1], Wann A.K.T*.[1], Thompson C.L.[1], Connelly J.T.[2], Knight M.M.[1]

[1] *Institute of Bioengineering and School of Engineering and Materials Science, Queen Mary University of London, Bancroft Road, Mile End, London E1 4NS, UK*
[2] *Institute of Bioengineering and Institute of Cellular and Molecular Sciences, Blizard Institute, Barts and The London School of Medicine and Dentistry, Queen Mary University of London, 4 Newark Street London, E1 2AT, UK*

*Address for correspondence:

Dr Angus Wann, School of Engineering and Materials Science, Queen Mary University of London, Mile End Rd, London, E1 4NS,UK. E-mail. a.wann@qmul.ac.uk Phone: +44 (0) 2078825368

Supplementary information

S1

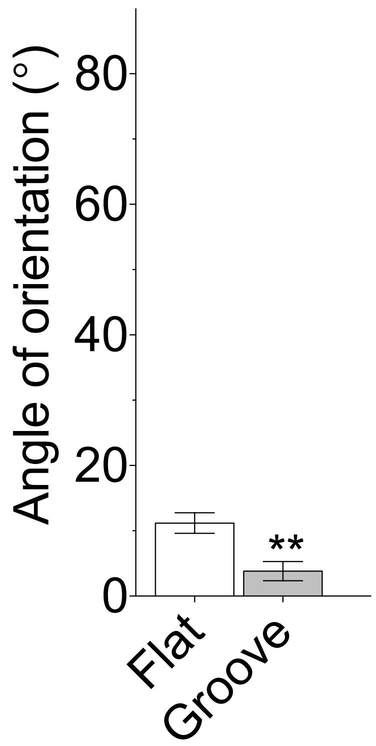

S2

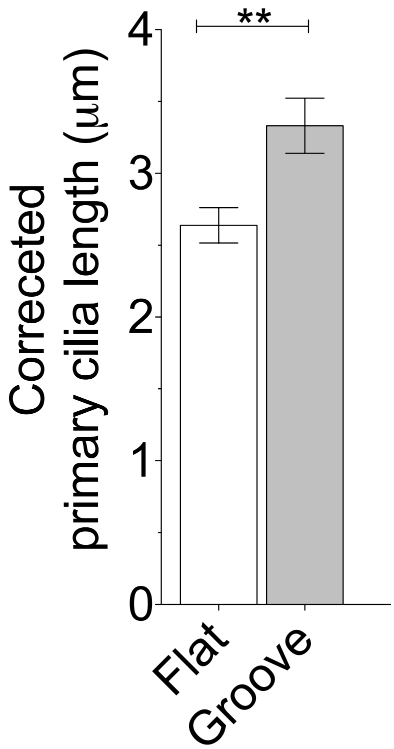

Supplementary figures S1 and S2

Angle of orientation relative to the horizontal substrate (S1) and corrected primary cilia length (S2) from analysis of confocal images. The orientation of each cilium was measured from confocal z stacks using the following equation which adjusts for the vertical distortion, relative to the substrate, caused by relatively poor axial resolution:

Angle of cilium orientation relative to horizontal (x-y) plane,

$$= \tan^{-1} \left(\left(\left((n-1) \times \Delta z \right) - \delta z - t \right) / L \right)$$

where n is the number of confocal sections in which the cilium appeared, L is the projected length measured by Image J from the maximum projection image, Δz is the confocal z-step size, t is the thickness of a cilium estimated at 0.2 μm and δz is the limit of resolution based on the full width half maximum (FWHM) of the point spread function corresponding here to a value of 0.5 μm.
On grooved surfaces there is a slight change in the orientation such that cilia angle (4°) is statistically significantly different to that measured for cells cultured on flat surfaces (11°) (S1) ($p<0.0001$, unpaired students t-test). Corrected cilia length is calculated using trigonometry assuming the cilia to be straight. Whilst there is a significant flattening of cilia on grooved topography once this is corrected for, cilia length increases with topography are still statistically significant (S2).
Error bars indicate S.E.M. ** indicates significant differences such that $p < 0.01$ for Mann-Whitney U tests.

S3

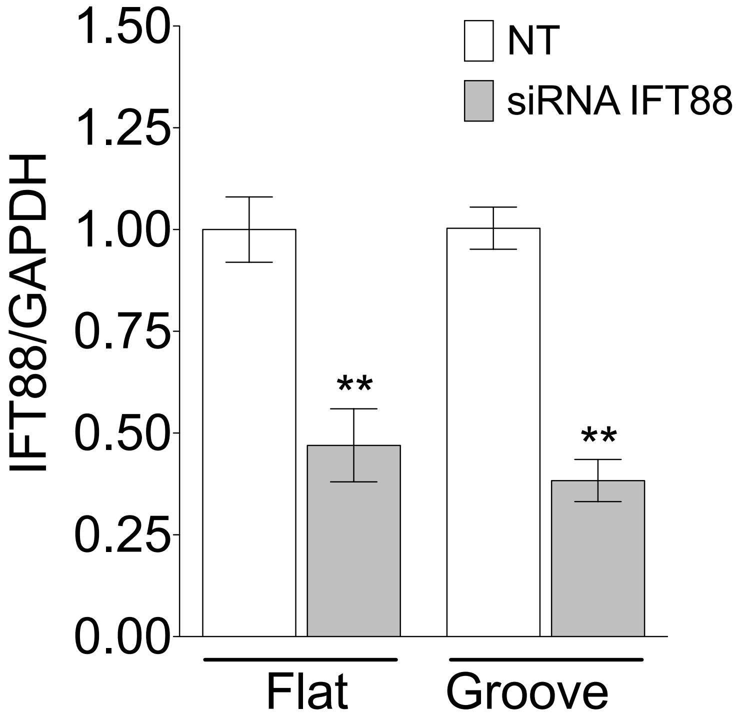

Supplementary figure S3

qPCR indicates 50-60% knock-down of IFT88 mRNA by siRNA pool targetted to IFT88 . Error bars indicate S.E.M.

** indicates significant differences such that p value < 0.01 for unpaired student's t-tests.
